# PD127 A Systematic Review Of The Clinical Effectiveness Of Recombinant Zoster Vaccine For Preventing Herpes Zoster In Adults

**DOI:** 10.1017/S0266462324003647

**Published:** 2025-01-07

**Authors:** David Byrne, Emma Reece, Orla Jenkins, Aoife Bergin, Carol McLoughlin, Joan Quigley, Conor Teljeur, Patricia Harrington, Máirín Ryan

## Abstract

**Introduction:**

The herpes zoster (HZ) virus is associated with significant morbidity. Its incidence and severity are higher among older adults and immunocompromised individuals. This systematic review assessed the clinical efficacy and effectiveness of recombinant zoster vaccine (RZV) for the prevention of HZ and associated complications in adults at least 50 years of age and in adults (≥18 years) at increased risk of HZ.

**Methods:**

Electronic searches restricted to between 2008 and July 2023 were conducted in Embase, MEDLINE, the Cochrane Library, and clinical trial registries. Two reviewers independently screened articles and extracted data. The review adhered to the PRISMA reporting guidelines. Quality appraisal was assessed using version two of the Cochrane risk-of-bias tool for randomized trials tool and the Risk of Bias in Non-Randomized Studies - of Interventions tool. Meta-analysis was undertaken using Cochrane methodology, with preference given to random effects meta-analysis because of study heterogeneity.

**Results:**

Twelve RCTs and five cohort studies were identified. Vaccine efficacy was defined as one minus the incidence rate ratio, multiplied by 100. For the general population, vaccine efficacy was 92 percent (n=29,311 individuals) and vaccine effectiveness was 70 percent (n=43,990,671 individuals). Based on one trial, vaccine efficacy in the general population (aged ≥50 years) waned from an initial 97.7 percent to 73.2 percent by year 10. Two RCTs reported vaccine efficacy for those at increased risk: 68.2 percent in hematopoietic stem cell transplant recipients and 87.2 percent in those with hematological malignancies. Secondary analyses were limited by sample size.

**Conclusions:**

There is clear, consistent evidence that RZV is effective in reducing HZ incidence. Although the vaccine is effective in those who are least 18 years of age and are at increased risk of HZ, efficacy may be lower compared with a general population aged at least 50 years. Secondary analyses (age subgroups, HZ complications, and HZ-related hospitalizations) were limited by small sample size, leading to inconclusive results.

